# Ultrafast switching of a metasurface quasi-bound state in the continuum via transient optical symmetry breaking

**DOI:** 10.1038/s41377-025-01885-z

**Published:** 2025-07-08

**Authors:** Giulia Crotti, Andrea Schirato, Olesiya Pashina, Olga Sergaeva, Mihail Petrov, Costantino De Angelis, Giuseppe Della Valle

**Affiliations:** 1 Dipartimento di Fisica—Politecnico di Milano, Milano, Italy; 2 Department of Physics and Astronomy, Rice University, Houston, TX USA; 3 School of Physics and Engineering, ITMO University, Saint Petersburg, Russia; 4 Dipartimento di Ingegneria dell’informazione, University of Brescia, Brescia, Italy; 5 Istituto Nazionale di Ottica, Consiglio Nazionale delle Ricerche, Brescia, Italy; 6 Istituto Nazionale di Fisica Nucleare—Sezione di Milano, Milano, Italy

**Keywords:** Nanophotonics and plasmonics, Ultrafast photonics

## Abstract

In photonic structures, bound states in the continuum (BICs) have recently attracted huge interest in both fundamental and applied research. Quasi-BIC leaky modes resulting from in-plane symmetry breaking in metasurfaces are particularly relevant to applications, due to their high quality factor, which scales as the squared inverse of the asymmetry parameter. Here, we theoretically propose an innovative approach to switch on quasi-BICs on sub-picosecond timescales via optically induced symmetry breaking in semiconductor metasurfaces. The desired effect is granted by exploiting the spatial inhomogeneities in the distribution of photo-excited hot carriers at the single meta-atom nanometric scale. In our simulations, the quasi-BIC state manifests itself as an ultra-sharp dip in transmission, emerging upon pump arrival, and disappearing completely within the carriers’ diffusion timescale. Our strategy allows to envision reconfigurable platforms with switchable high-Q resonances, with ultrafast recovery beyond the limits of carrier relaxation, typical of previous approaches.

## Abstract (web summary)

Nanoscale optical-symmetry breaking in all-dielectric meta-atoms photoexcited by ultrashort light pulses produces BIC-to-quasi-BIC switching. An ultrasharp resonance, absent in unperturbed conditions, is transiently photoinduced, vanishing within a few picoseconds.

## Graphical abstract

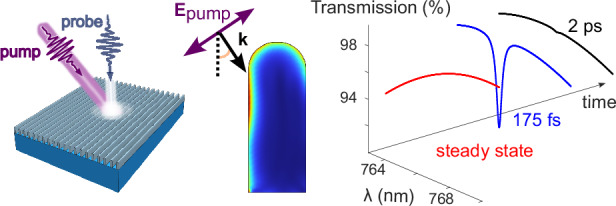

## Introduction

Bound states in the continuum (BICs) are solutions of wave equations, corresponding to spatially localised states, whose energy is embedded in the continuum of radiative modes^1,2^. Electromagnetic BICs in systems such as waveguide arrays, photonic crystals and metasurfaces are widely investigated on account of their interesting properties. From a theoretical standpoint, the description of their topological nature^3^ has opened new perspectives in the field of topological photonics^4–7^. As for applications, BIC-based mechanisms have been implemented for lasing^8–12^, sensing^13–16^, filtering and controlling light^17–19^, and more^20,21^.

Several of these approaches are based on the fact that systems supporting BICs are intrinsically high quality factor resonators: indeed BICs, being completely decoupled from radiation, can be conceptualised as non-excitable resonances with infinite quality factor, or vanishing linewidth. Extremely sharp resonances become excitable whenever leaking channels are opened for the bound mode. This can be done e.g. for the particular class of BICs whose existence is protected by rotational or reflection symmetry^1^. Upon the breaking of such symmetry, for example via slight modifications of the structure geometry, BICs turn into quasi-BIC modes coupled to radiative waves, with quality factor scaling as the squared inverse of the asymmetry parameter^22^. In scattering, the excitation of a quasi-BIC mode manifests as a resonance in the transmission or reflection spectrum; accordingly, with a slight abuse of notation, we refer to these features as to 'quasi-BIC resonances' to indicate their origin.

Thus, tuning the degree of symmetry breaking allows to tailor the optical response of the system, to obtain, for example, high sensitivity sensors, or custom narrow-band filters for visible light and THz radiation. One could even envisage to modulate the polarization of light, thanks to the fact that, depending on the topological charge of the BIC, breaking in-plane symmetries can generate circularly polarized resonances^4,5^. Furthermore, in recent years, several studies have demonstrated active control of quasi-BIC resonances by proposing a variety of modulation mechanisms and structural designs for dynamic applications^23–33^.

In this context, an intriguing advancement is represented by the capability of triggering the conversion from a proper BIC state to a quasi-BIC mode and vice versa, by actively inducing and controlling the symmetry breaking. Up to now, only a few papers have presented strategies for this challenging task^34–38^, which are mainly based on the same rationale. The employed platforms are (either semiconductor or metallic) hybrid metasurfaces, designed to operate in the THz regime. The elementary meta-atoms are asymmetric in unperturbed conditions, the resonators being constructed by two different materials. One of them (e.g. graphene or photoconductive silicon) has a conductivity which is reconfigurable by either electrical, thermal or optical means. Thus, by regulating the intensity of the stimulus, the asymmetry can be either enhanced or reduced, and even destroyed, so that the quality factor of the quasi-BIC can be tuned to be higher and higher and, eventually, a proper switching to a BIC (non-resonant) state can be achieved.

However, this approach relies on the capability of precise manufacturing of composite, slightly asymmetric structures at the unit cell level: indeed, it has been applied only to THz metasurfaces, with a relatively large typical size of the meta-atoms of the order of tens of microns. Scaling this approach to visible or infra-red light would be extremely challenging with the available fabrication techniques. On the other hand, an electrically reconfigurable, asymmetric *environment* in a metasurface unit cell has recently been proved effective to modulate a quasi-BIC resonance at optical wavelengths^39^. Nevertheless, the concept still relies on elaborated manufacturing techniques; besides, the switching speed remains comparatively low (~18 ms) with respect to the ones achievable with an all-optical procedure. Moreover, another challenge is represented by the addressability of the single elements that, in the unit cell, can be modulated to regulate the asymmetry of the structure. Therefore, a change of paradigm becomes necessary to achieve ultrafast switching between BICs and quasi-BICs at the nanoscale.

In this paper, we theoretically propose a new strategy to this aim. It is based on the concept of photo-induced breaking of the optical symmetry in a semiconductor metasurface, operating in the near-infrared range of the electromagnetic spectrum. The rationale is explained as follows. We devised a metasurface that, in unperturbed conditions, supports a symmetry-protected BIC at the Γ-point of the first Brillouin zone. The absorption of intense femtosecond (fs) laser pulses in the dielectric meta-atoms generates a population of nonequilibrium, 'hot', electron-hole pairs, which in turn causes a transient modulation of the semiconductor permittivity. Spatial inhomogeneities in the carrier distribution, due to asymmetric absorption patterns^40^, entail a sub-picosecond breaking of an in-plane symmetry, hence the ultrafast switching of a quasi-BIC state.

To quantitatively describe this scenario, we employ a semi-classical model for the inhomogeneous photoexcitation and ensuing third-order nonlinearity, and combine it with full-wave numerical simulations for the electromagnetic problem, to extract the transient optical response of the metasurface. Our results show that, upon interrogation of the structure with a probe pulse, the quasi-BIC switching is manifested as an extremely sharp resonance in the transmission spectrum, appearing almost instantaneously after pump arrival and disappearing again when the optical symmetry is restored—i.e., on the carriers’ diffusion timescale (~2 ps).

The swift recovery of the system’s optical response (the restoration of a true BIC) demonstrates that this approach could open new perspectives in the more general context of ultrafast active modulation of metaphotonic structures by all-optical means^41^. Indeed, it allows to overcome the intrinsic speed limits posed by the timescale of carrier relaxation (of the order of tens of picoseconds), typical of previous studies.

## Results

**Fig. 1:**
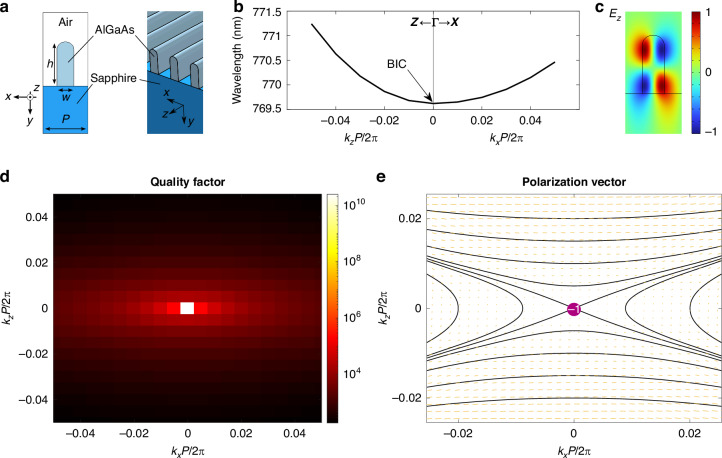
**Nanowires AlGaAs metasurface**. **a** Left. Cross-section of the unit cell of the simulated metasurface. The geometrical parameters are *P* = 400 nm, *w* = 150 nm and *h* = 400 nm. Right. 3D scheme of the metasurface. **b** The investigated TE-like band in the vicinity of the Γ point. The right (left) part of the panel refers to a path from the Γ point to the X (Z) point in the First Brillouin Zone. **c** Normalized *E*_*z*_ component of the BIC mode at Γ point. **d** Quality factor of the resonances of the investigated band. The colour scale is logarithmic. **e** Far-field polarization (on the downward or transmission direction) of the investigated band across the reciprocal space. The vector field and streamlines represent the major axis of the far field polarization ellipse; the length of the line segments is proportional to the amplitude of the radiated field. At the Γ point, the vortex centre corresponds to a BIC with topological charge − 1

The first designed metasurface is a 1D-periodic arrangement of Al_.18_Ga_.82_As nanowires on top of a transparent sapphire substrate (refractive index *n*_sub_ = 1.75). A scheme of the unit cell cross-section and a 3D picture of the structure are depicted in Fig. 1a, where the structural parameters are also defined.

Notice that, according to the notation in Fig. 1a, the *y* direction corresponds to the vertical axis, i.e., the direction normal to the interface plane *xz*, and translational invariance is enforced along the *z* axis. As such, the in-plane symmetries of this geometry belong to the *C*_2v_ group, that is, the structure is invariant both under a 180^∘^ rotation around the *y*-axis (${C}_{2}^{y}$), and under two mirror reflections about the *xy*- and *yz*- vertical planes (*σ*_v_(*xy*) and *σ*_v_(*yz*), respectively). This grants the existence of robust BICs at high-symmetry (i.e., ${C}_{2}^{y}$-invariant) wave vectors^3^. In other words, at the Γ point, every even eigenstate of the structure under ${C}_{2}^{y}$ is completely decoupled from radiative waves, which are odd under the same symmetry^1^.

Indeed, for 1D and 2D-periodic systems such as metasurfaces, below the diffraction limit and above the light line, eigenmodes with a specific in-plane wave vector **k**_∥_ are coupled, in general, with plane waves propagating either upwards (*u*) or downwards (*d*) with respect to the slab, having the same (in-plane) **k**_∥_ and a defined polarization state **c**^u,d^(**k**_∥_)^3–6^. The far-field polarization is a suitably defined projection ${{\bf{c}}}^{{\prime} \text{u,d}}({{\bf{k}}}_{\parallel })$ of **c**^u,d^(**k**_∥_) onto a plane parallel to the slab. In reciprocal space, BICs manifest themselves as vortex centres in the direction of the main axis of the far-field polarization ellipse. The associated integer topological charge counts how many times the far-field polarization ellipse winds along a path enclosing the singularity, travelled counter-clockwise. For more details, see section S1 of the Supporting Information document (SI).

We focused on a photonic band whose modes are, in the array region, polarized parallel to the interface plane, along the *z* axis (TE-like), with energies just below the AlGaAs bandgap (having energy *E*_g_ ≃ 1.65 eV, i.e., *λ*_g_ ≃ 750 nm). In this region, the permittivity is purely real. The band belongs to the *A* representation of the ${C}_{2}^{y}$ group, thus a BIC can be found at Γ point, **k**_∥_ = (*k*_*x*_, *k*_*z*_) = (0, 0). A portion of the calculated photonic band is depicted in Fig. 1b, whereas the simulated electric field *E*_*z*_ of the BIC mode inside the unit cell is shown in Fig. 1c. We can see that this eigenmode corresponds to a true BIC by examining the quality factor and the far-field polarization topology of the photonic band, shown in Fig. 1d,e, respectively. The first colormap, in logarithmic scale, shows the calculated radiative quality factor of the resonances in the band; it diverges as the Γ point is approached. In Fig. 1e, the streamlines and line field represent the direction of the major axis of the polarization ellipse ${{\bf{c}}}^{{\prime} \text{d}}({{\bf{k}}}_{\parallel })$, evaluated on a *xz*-plane in the substrate, far below the metasurface. The length of each line segment is proportional to the amplitude of the mode’s radiative component. The Γ point is a vortex centre, corresponding to a topological charge *q* = −1. The upward far-field polarization (not shown here) has the same topological structure around **k**_∥_ = **0**, with ${{\bf{c}}}^{{\prime} \text{u}}({{\bf{k}}}_{\parallel })$ also vanishing there, thanks to ${C}_{2}^{y}$ symmetry^3^.

These results were obtained by numerically solving Maxwell's equations in the eigenvalue formulation with commercial software (COMSOL Multiphysics^42^). Details on the numerical implementation can be found in the Methods and in section S2 of the SI.

For systems such as the ones described above, it has been shown that the breaking of ${C}_{2}^{y}$ via alterations of the in-plane geometry entails that the BIC splits into a pair of circularly polarized states (C-points) of opposite helicity in the vicinity of Γ^4–6^. The mode at Γ becomes radiative, while the vorticity structure of Fig. 1e is preserved, as the C-points also represent singularities in the polarization field with half-integer topological charge (−1/2)^4–6,43^. Following a similar rationale, we performed a proof-of-concept simulation to demonstrate that an asymmetric permittivity perturbation in the otherwise ${C}_{2}^{y}$-symmetric metasurface produces the same effect.

**Fig. 2:**
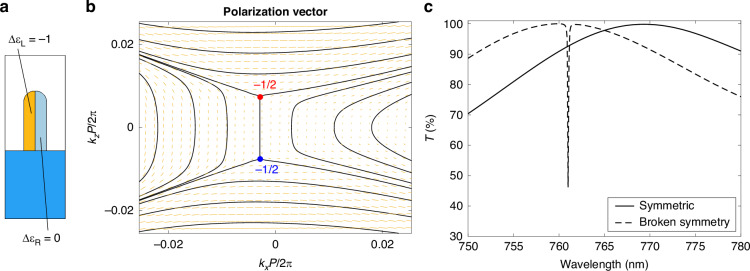
**Broken optical symmetry**. **a** Scheme of permittivity modulation. **b** Far-field polarization (in transmission) in the broken symmetry case. Red (blue) point represents a circularly polarized resonance with positive (negative) helicity, corresponding to a vortex centre for the polarization with topological charge −1/2. **c** Transmission of the metasurface in static (solid trace) versus perturbed conditions (corresponding to broken symmetry, dashed trace). The spectral resolution of the simulations is 0.1 nm

As illustrated in Fig. 2a, we divided the wire in two domains, setting a modulation Δ*ε*_L_ = −1 of AlGaAs permittivity on the left half and leaving the right part unperturbed, Δ*ε*_*R*_ = 0. The eigenfrequency analysis yields the results summarized in Fig. 2b in terms of the ${{\bf{c}}}^{{\prime} \text{d}}({{\bf{k}}}_{\parallel })$ (downward) far-field polarization.

As expected, the symmetry breaking induced by the permittivity perturbation leaves the global topological structure unaltered, with the total charge conserved. Two C-points of opposite helicity have appeared and, importantly, the singularity at Γ is no longer present. The mode is now radiative, with a well-defined linear polarization along the *z*-axis in the far field. A similar pattern is observed when analyzing ${{\bf{c}}}^{{\prime} \text{u}}({{\bf{k}}}_{\parallel })$: the upward propagating radiation (not shown here) is also non-vanishing and linearly polarized along *z*.

The purely optical symmetry breaking caused by inhomogeneous permittivity variation thus turns the BIC into a quasi-BIC leaky mode, coupled to both upward and downward propagating, out-of-plane polarized plane waves. To verify this, the unperturbed versus perturbed optical response of the system is investigated in two scattering simulations. In each of them, the incident plane wave is linearly polarized along *z* and impinging from air onto the metasurface at normal incidence, so that its parallel wave vector **k**_∥_ vanishes. This is consistent with the goal of exciting the quasi-BIC leaky mode at Γ in the perturbed system. The obtained transmission spectrum is plotted in Fig. 2c as a function of wavelength, in the unperturbed/symmetric (solid line) and perturbed/broken-symmetry case (dashed line).

The unperturbed transmission is the one of a non-resonant system, and is approximately 100% at ~769 nm, corresponding to the BIC wavelength. Instead, the extremely narrow (~1 nm) dip in transmission at 761 nm, appearing in perturbed conditions, is precisely the quasi-BIC resonance originated from symmetry breaking. In reflection (not shown here), it is manifested as a sharp peak. The blue-shift is caused by the negative Δ*ε*_*L*_, i.e., by a reduction of the effective index of the slab.

Having demonstrated that an *optical* breaking of the ${C}_{2}^{y}$ symmetry allows to obtain results as the ones yielded by geometrical symmetry-breaking, we propose how to induce a realistic, asymmetric permittivity modulation, similar to the one of Fig. 2a, by all-optical means. We thus defined a complete model to study the quasi-BIC evolution on an ultrafast timescale. Illumination of a direct semiconductor with an ultrashort, intense pump pulse with energy above bandgap entails the generation of electron-hole pairs through linear absorption. These energetic “hot” carriers preside over the permittivity modulation via few diverse effects. Their diffusion and relaxation towards the lattice (mainly through non-radiative recombination processes with phonon emission) regulate the ultrafast transient response of the system. These phenomena are often described by considering the carriers and the lattice as coupled thermal reservoirs, within the framework of the Two Temperature Model (2TM). This is a rate equation system in the variables *N*(*t*), the pairs number density, and Θ_L_(*t*), the lattice temperature, which are considered as functions of time only. In the classic 2TM formulation, therefore, spatial transients are neglected.

To include them, we developed an Inhomogeneous version of the model (I2TM), which is the semiconductor analogue to the one employed for metals in some of our previous works^40,44^. It reads as follows:

1 $$\begin{array}{lll}\frac{\partial N({\bf{r}},t)}{\partial t}\;\;=\;{R}_{{\rm{abs}}}({\bf{r}},t)-\nabla \cdot \left(-D\nabla N({\bf{r}},t)\right)-\frac{N({\bf{r}},t)}{{\tau }_{{\rm{rec}}}}\\ \frac{\partial \Theta L({\bf{r}},t)}{\partial t}\;=\;\frac{{E}_{{\rm{g}}}}{{c}_{{\rm{L}}}}\frac{N({\bf{r}},t)}{{\tau }_{{\rm{rec}}}}-\nabla \left(-\frac{{\kappa }_{{\rm{L}}}}{{c}_{{\rm{L}}}}\nabla \Theta _{\rm{L}}({\bf{r}},t)\right)\end{array}$$

The carrier density and lattice temperature are now conceived as functions of both time and space, and the model is solved locally, taking diffusive processes into account. Moreover, the source term *R*_abs_ in the first equation is also space dependent and proportional to the pump absorption *ρ*(**r**, *λ*_pump_). This is defined in the wire as the density of dissipated electromagnetic power, normalized to the incident power; in our effective 2D system, it has the dimensions of a surface density, [m^−2^], and is extracted from a full-wave simulation of the system response to the optical pump. Thus,

2 $${R}_{{\rm{abs}}}({\bf{r}},t)=A\rho ({\bf{r}},{\lambda }_{{\rm{pump}}})g(t)$$

with *A* a constant incorporating geometric quantities and pump parameters, and *g*(*t*) the temporal envelope of the pulse.

Other relevant quantities are the carriers’ diffusion coefficient *D*, the timescale of trap-assisted recombination *τ*_rec_, the lattice heat capacity and thermal conductivity, *c*_L_ and *κ*_L_, respectively. More details on the numerical implementation are included in the Methods section and in section S2 of the SI.

**Fig. 3:**
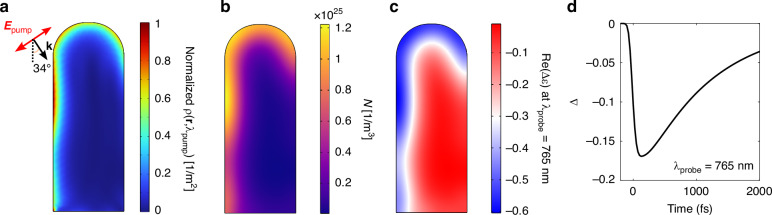
**Photo-induced symmetry breaking**. **a** Absorption field *ρ*(**r**, *λ*_pump_), normalized to its peak value inside the nanowire. The inset shows the excitation conditions: the angle of incidence θ = 34^∘^ and the in-plane pump polarization. The pump is centred at *λ*_pump_ = 400 nm, has a temporal full width at half maximum (FWHM) of *τ* = 100 fs and a low fluence *F* ~ 40 *μ*J cm^−2^. **b** Electron-hole pair density *N*(**r**, *t*) at *t* = 100 fs. **c** Real part of the permittivity variation Δ*ε* at *λ*_probe_ = 765 nm and time delay *t* = 100 fs. **d** Degree of asymmetry Δ at *λ*_probe_ = 765 nm as a function of time delay

Figure 3a shows *ρ*(**r**, *λ*_pump_), normalized to its peak value in the wire, for the simulated excitation conditions: the pump pulse, centred at *λ*_pump_ = 400 nm and with a full width at half maximum of 100 fs, is polarized in the vertical plane and impinges at 34^∘^ with respect to the normal direction, as sketched in the inset on the left. This configuration maximises the degree of symmetry breaking, as apparent by the distribution of the hot-spots, mainly concentrated in the left part of the wire (see also section S3 of the SI). The calculated distribution of hot carriers *N*(**r**, *t*) at *t* = 100 fs after the pump peak is depicted in Fig. 3b, and reflects the inhomogeneity of the absorption field.

As mentioned, the presence of free carriers and the increase in lattice temperature cause transient broadband modifications of permittivity Δε^45–47^. In the model, we took into account Drude and band filling effects on Δε, ascribable to the electron-hole pairs, and thermo-optic variations due to the lattice. However, in this discussion we will focus only on carriers’ contributions, as thermo-optic phenomena happen on longer timescales (tens of ps), and are comparatively weaker for low pump fluences as the one simulated here (*F* ~ 40 *μ*J cm^−2^).

Specifically, the formation of electrons and holes plasmas opens channels for intraband optical transitions: in this respect, AlGaAs permittivity is modified by a Drude-like term, Δε_D_(**r**, *t*, *λ*_probe_). On the other hand, band filling accounts for a modification of the interband transitions, representing a saturation of the absorption channels due to Pauli exclusion principle^48^. In the spectral range of the studied BIC, i.e., around 770 nm, the dominating phenomenon is band filling^47^, which entails a purely real contribution Δε_BF_(**r**, *t*, *λ*_probe_) for probe wavelengths longer than *λ*_*g*_ = 750 nm. Instead, Δε_D_ also features a small imaginary part, with values up to ~ i0.002 for all the investigated probe wavelengths. All details on the semiclassical solid-state calculations to retrieve Δε can be found in section S2 of the SI.

Figure 3c shows the real part of the total Δε, sum of the carriers (Δε_D_ + Δε_BF_) and thermo-optic (Δε_TO_) terms, for *λ*_probe_ = 765 nm and evaluated at a time *t* = 100 fs after the arrival of the pump pulse peak. Re(Δε) has maxima (in absolute value) concentrated in the hot-spots on the top and left part of the wire. The bottom right region features instead up to two orders of magnitude smaller ∣Re(Δε)∣ values. Again, the asymmetry at such early stages after arrival of the exciting pump pulse is a direct consequence of the inhomogeneities in the *N*(**r**, *t*) field.

In analogy to what presented in Fig. 2, the degree of symmetry breaking can be quantified by defining Δ(*t*, *λ*_probe_) as the contrast of permittivity across the nanowire, i.e., as the difference between the spatial average of Re(Δε) on the left and right domains: $\Delta ={ < \text{Re}(\Delta \varepsilon ) > }_{L}-{ < \text{Re}(\Delta \varepsilon ) > }_{R}$. This is depicted as a function of time in Fig. 3d for *λ*_probe_ = 765 nm. The steep descent of Δ is simultaneous to the arrival of the pump pulse; the peak value, corresponding to maximal asymmetry, is Δ ≃ −0.17, reached at ~125 fs. Δ is subsequently halved within ~1 ps, as a consequence of carrier diffusion, which entails a gradual symmetry restoration.

Finally, we calculated the transient optical response of the system, perturbed by the modelled carrier distribution, via full-wave scattering simulations. A typical figure of merit in pump-probe spectroscopy is the differential transmission Δ*T*/*T* = (*T* − *T*_0_)/*T*_0_, with *T* the transmission as a function of the probe wavelength and time delay with respect to the pump peak, and *T*_0_ the corresponding transmission of the unperturbed sample.

**Fig. 4:**
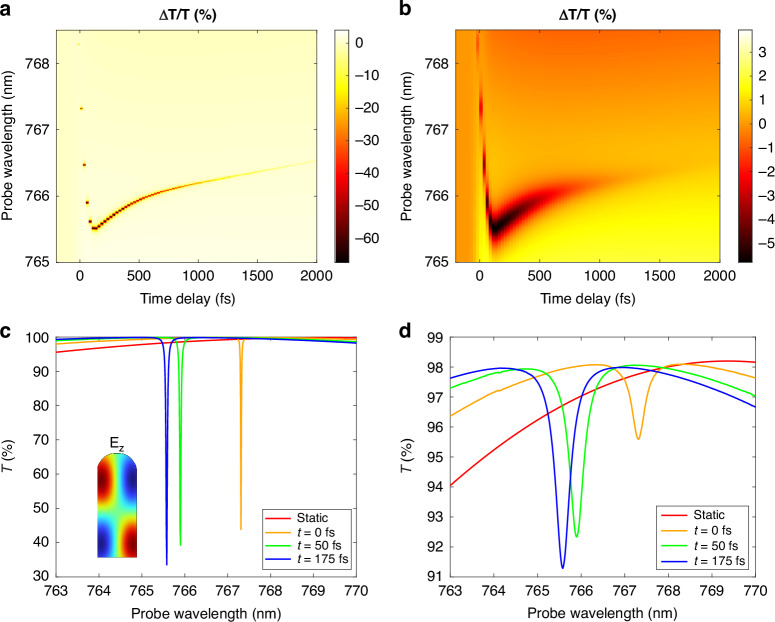
**Ultrafast quasi-BIC switching**. **a**, **b** Maps of the simulated Δ*T*/*T* without (**a**) and with (**b**) the additional losses Δε_add_ = i0.01. The temporal and spectral resolution of the simulations are 25 fs and 0.01 nm, respectively. **c**, **d** Transmission spectra at selected time delays, obtained from the same simulations as the respective panels above. The inset in (**c**) shows the *E*_*z*_ electric field at *λ*_probe_ = 765.58 nm and *t* = 175 fs, corresponding to the minimum of the blue curve

The computed Δ*T*/*T*, expressed in percentage, is shown in Fig. 4a. In addition, Fig. 4c includes the corresponding transmission curves in static versus perturbed conditions at selected time delays. An ultra-narrow feature (~0.05 nm) is manifest already at *t* ≃ 0 fs, almost simultaneous to the pump arrival, at 767.3 nm, close to the BIC spectral region. The resonance is blue-shifted and slightly broadened on a timescale of ~ 100 fs, then almost completely disappears within 2 ps. The switching is particularly evident when comparing the static transmission of the sample (red curve in Fig. 4c) with the ultrafast response after pump arrival (orange, green and blue curves). Moreover, the electric field in the nanowire at resonance (see inset in Fig. 4c, evaluated for *λ*_probe_ = 765.58 nm, *t* = 175 fs) corresponds to the BIC pattern (Fig. 1c).

Notice that the swift disappearance of the resonance (or equivalently, the decay of the asymmetry parameter Δ of Fig. 3d) cannot be attributed to the carriers’ lifetime, since trap-assisted recombination takes place on a much longer timescale^45–47^, described in the I2TM by the parameter *τ*_REC_ = 8 ps. Indeed, consistently, the simulation predicts a residual pump-probe signal at 2 ps, whose low intensity is due to the fact that the optical response is again non-resonant, as in the steady state.

Figure 4 (panels b,d) also shows the results of a further simulation, in which we employed an additional term Δε_add_ = i0.01 for the imaginary part of the permittivity, to mimic scattering losses due to imperfections of typical physical samples, or residual absorption below the bandgap. This contribution is spatially and temporally uniform and applied for all probe wavelengths. Thus, Im(Δε) ≃ 0.01, i.e., it is increased by an order of magnitude with respect to the first configuration. This second simulation is expected to yield more realistic predictions for experiments. In this case, we observe the same peculiar dynamics, aside from a broadening of the transmission dip to a maximum of ~0.5 nm. Here, the instantaneous appearance of the feature, as well as its blue-shifting and broadening, can be appreciated even more readily.

Another effect seen in Fig. 4b–d is a reduction of the contrast between resonant and non-resonant transmission, hence of the pump-probe signal. Notice that this is consistent with the fact that we modelled all additional losses as *absorption* losses: resonances with high quality factors such as the one presented here, whose field is strongly localized within the nanoresonator, are extremely sensitive to dissipation in the material. In the 'Discussion' section we provide a list of strategies for possibly enhancing such contrast, if required to address specific applications.

In any case, the results of the two calculations in Fig. 4, exhibiting the same trend, indicate indeed that photo-induced inhomogeneities allow for switching on a quasi-BIC resonance, which manifests itself as soon as the symmetry is broken, upon pump arrival. The hot carriers, generated non-uniformly in space, are responsible for the transient, inhomogeneous Δε. Its real part increases in module as the carrier density *N* builds up—causing, in turn, the resonance blue-shift within the first hundred of fs, i.e., the pump temporal FWHM. At the same time, as a consequence of the growing asymmetry produced by the contrast of Re(Δε) across the nanowire (Fig. 3d), the dip is broadened. Then, the carriers diffusion restores the symmetry on the picosecond timescale, leading to the disappearance of the quasi-BIC state. The comparison with the evolution of the asymmetry parameter Δ also supports this interpretation of the ultrafast dynamics. In view of these results, we think that the effect should be sizable and observable in experiments if sufficient temporal (~50 fs) and spectral resolution (~0.5 nm) can be provided.

**Fig. 5:**
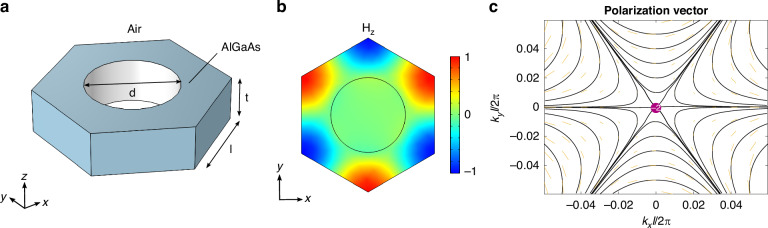
**AlGaAs slab metasurface**. **a** Unit cell of the hexagonal grating. The geometric parameters are *d* = 194 nm, *l* = 200 nm, *t* = 95 nm. The slab is free-standing and embedded in an air environment. **b** Normalized *z*-component of the magnetic eigenfield at Γ (cross-section taken at the central *z* = 0 plane). **c** (Downward) far-field polarization of the relevant photonic band. At *Γ*, the vortex centre corresponds to a BIC with *q* = −2

Interestingly, our approach has general validity and is not limited to the AlGaAs nanowire metasurface detailed above. In the following, we illustrate an additional metasurface design, aimed at demonstrating the same approach in a different configuration, for a BIC with higher topological charge, *q* = −2. It is a free-standing Al_.18_Ga_.82_As slab, with circular holes, constituting a 2D-periodic lattice with a hexagonal unit cell (see Fig. 5a). The chosen photonic band is TE-like (in the metasurface region, its eigenmodes are polarized in-plane), and, at *Γ*, belongs to the *B*_1_ representation of the point group *C*_6v_, whose elements represent the in-plane symmetries of the structure^5^.

This is seen by inspecting *H*_*z*_, the *z*-component of the magnetic field corresponding to the eigensolution of Maxwell’s equations at Γ (Fig. 5b). This mode is a symmetry-protected BIC^3,5^, as evident also from the far-field polarization: see the streamlines and line field in Fig. 5c, representing the direction of the main axis of ${{\bf{c}}}^{{\prime} \text{d}}({{\bf{k}}}_{\parallel })$, and featuring a vorticity structure around **k**_∥_ = (0, 0). The singularity has a topological charge *q* = −2, since the polarization vector winds twice along a curve enclosing Γ. The energy eigenvalue of this mode corresponds to a wavelength of ~772 nm, i.e., this photonic band belongs to a spectral region close to the one studied for the nanowires metasurface.

We performed a full-wave simulation for the pump excitation, to extract the absorption pattern *ρ*(**r**, *λ*_pump_)—see the 'Methods' for further details. We kept the same temporal FWHM for the pump pulse as before (*τ*_FWHM_ = 100 fs), while we increased the pump fluence (*F* = 70 *μ*J cm^−2^). This value still entails a moderately low level of photo-excitation^46^. In this case, the pump illumination conditions were chosen specifically to break the *C*_3_ symmetry, while maintaining *C*_2_. This is sufficient to turn the at-Γ BIC into a quasi-BIC^5^: accordingly, the pump was set at normal incidence, with electric field polarized along the *y*-axis. Secondly, we solved the I2TM and obtained the pair density and lattice temperature distributions *N*(**r**, *t*), Θ_L_(**r**, *t*). We focused on a specific time delay after pump arrival, *t* = 300 fs. It belongs to a temporal window in which carriers, generated in hot spots on the upper part of the structure, have already diffused in the bulk of the slab; however, the delay is sufficiently short to ensure that homogenisation (hence, symmetry restoration) has not been attained yet.

**Fig. 6:**
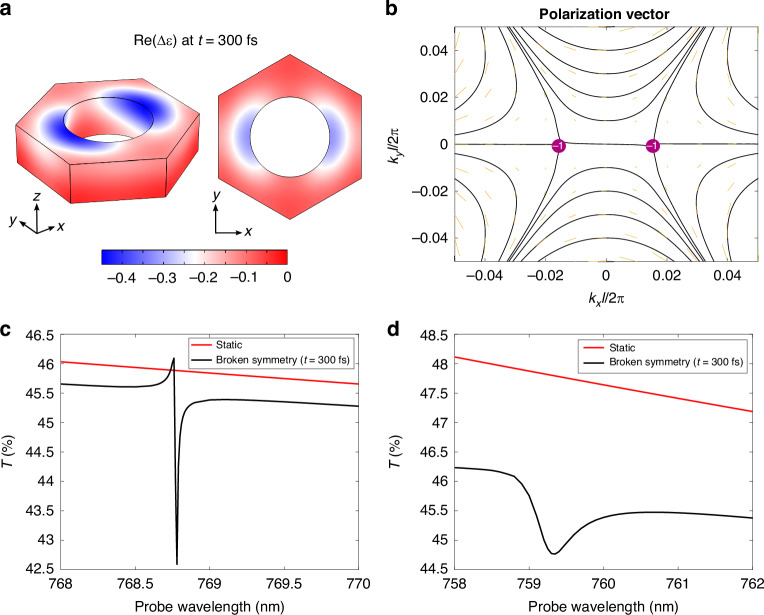
**Broken symmetry and quasi-BIC switching**. **a** Left: Δε pattern on the slab surface in the unit cell at *t* = 300 fs and *λ*_probe_ = 768.5 nm. Right: Δε at the same time delay and probe wavelength, cross-section on the central (*z* = 0) plane. **b** Downward far-field polarization in the perturbed (broken symmetry) case. The at-Γ BIC has split into two topological defects with charge *q* = −1. **c** Simulated transmission of the metasurface in the static (red curve) versus perturbed case (black curve). **d** Same as in panel c), for the additional simulation considering extra losses Δε_add_ = i0.01 and higher pump fluence, *F* = 500 *μ*J cm^−2^

We computed the permittivity variation Δε as explained above for the nanowires metasurface configuration (without the addition of fictitious losses, since, in this case, we are interested in a proof-of-concept simulation rather than a quantitative prediction of experiments). The real part of Δε, evaluated at 300 fs for a probe wavelength *λ*_probe_ = 768.5 nm, is represented in Fig. 6a, both on the slab surface and on a cross-section taken at the central plane (*z* = 0). The pattern shows the expected *C*_3_ symmetry breaking.

Having set such Δε, we first performed an eigenfrequency study to understand how the far-field polarization is affected by the asymmetric permittivity perturbation. The *C*_3_ symmetry breaking reduces the at-*Γ* eigenmode to the *B*_1_ representation of the *C*_2v_ group. This entails that the *q* = −2 BIC in reciprocal space is split into two off-*Γ* topological defects, each of charge *q* = −1, to grant global charge conservation^3,5^. Indeed, this is what is observed when inspecting both the downward (Fig. 6b) and upward far-field polarization (Fig. S1a in the SI document, red curves): two singularities appear on the *k*_*x*_ axis.

This is consistent with previous studies^5^, in which geometric symmetry breaking was implemented. However, notice that the photo-induced perturbation also breaks the up-down mirror symmetry *σ*_*z*_ of the structure. This translates into the fact that the ${{\bf{c}}}^{{\prime} \text{d}}({{\bf{k}}}_{\parallel })$ and ${{\bf{c}}}^{{\prime} \text{u}}({{\bf{k}}}_{\parallel })$ are independent as complex fields over the reciprocal space^3^, albeit having the same topological structure. Thus, the *q* = − 1 defects illustrated in Fig. 6b only represent points where *downward* radiation is forbidden; they are not BICs^6^, since their position on the *k*_*x*_ axis does not correspond to the position of the *upward* vector field singularities. A more thorough discussion on this topic is presented in section S1.2 of the SI document.

Nevertheless, the at-Γ state is now radiative, with linear polarization along the *x*-direction in far field. To demonstrate this, we performed a full-wave scattering simulation to probe the response of the system in static versus perturbed conditions. The probe pulse is an *x*-polarized plane wave, impinging at normal incidence onto the slab from above (i.e., from the same side as the pump pulse). The transmission spectra are shown in Fig. 6c. Also in this case, the unperturbed metasurface has the response of a nonresonant system, as the BIC cannot be excited by propagative waves. Instead, a sharp quasi-BIC resonance appears in the broken symmetry case, blue-shifted from ~772 nm to ~768.8 nm as a consequence of the negative permittivity variation.

These results indicate that, also in this configuration, photo-induced optical symmetry breaking yields the same effects produced by geometrical perturbations, in relation to both the far-field polarization topology and the switching of a quasi-BIC. Moreover, other possible scenarios for the efficient modulation and control of light can be envisioned: for example, the quality factor of the steady-state leaky modes at **k**_∥_ = (*k*_*x*_, 0), near Γ, would be dynamically enhanced by symmetry breaking, with an interesting temporal evolution determined by the diffusion of carriers.

The simulation above presents only a proof-of-concept, ideal scenario, since no extra losses are included. In a real experiment, the high quality factor and the relatively reduced contrast with respect to the non-resonant transmission are expected to make the quasi-BIC resonance difficult to observe. We show here that it is possible to solve both issues by pumping at higher fluences, thus increasing the permittivity modulation and hence the symmetry breaking. In turn, this allows to (i) broaden the resonance, and (ii) enhance the in-coupling of the incoming probe and the quasi-BIC mode, thus increasing the resonance contrast, so that additional losses cannot cancel the effect.

Figure 6d shows the static versus broken symmetry case, simulated with increased pump fluence *F* = 500 *μ*J cm^−2^ and extra losses Δε_add_ = i0.01. The quasi-BIC resonance corresponds to a drop in transmission that, in terms of Δ*T*/*T*, should produce a ~6% signal. This could be readily observable in measurements. Notice that the feature is more blue-shifted compared to Fig. 6c, as a consequence of the stronger permittivity modulation.

## Discussion

In summary, we proposed a new approach for ultrafast modulation of light with semiconductor metasurfaces, based on a symmetry-protected BIC that is transiently turned into a resonance via photo-induced symmetry breaking.

We first designed a 1D-periodic metasurface of AlGaAs nanowires on a sapphire substrate, with a symmetry-protected, at-Γ BIC in steady-state conditions. Our theoretical model, describing transient inhomogeneities of hot carriers generated at the nanoscale by fs-laser pulses in the visible, predicts the appearance of a sharp resonance in transmission under relatively low fluence (tens of *μ*J cm^−2^). This phenomenon is dictated by the photo-induced asymmetry in the structure, arising from the hot-carrier-mediated permittivity modulation at the band edge of AlGaAs^47^. The quasi-BIC resonance disappearance, due to diffusion and the subsequent restoration of homogeneity, is much faster than the complete relaxation of the carrier-lattice system, making this process exploitable in a truly reconfigurable device for all-optical modulation approaching THz switching rate^41^.

Moreover, we showed that our approach can be easily extended to more general BIC systems and to different kinds of symmetry breaking. In particular, we considered a free-standing AlGaAs metasurface membrane with 2D-periodic hexagonal lattice. This structure supports an at-Γ BIC with higher topological charge (*q* = −2) and presents a richer variety of topological phenomena related to the symmetry breaking^3,5^. Our simulations demonstrate the possibility to induce, by all-optical means, the splitting of the topological charge, corresponding to the switching from BIC to quasi-BIC. This can be accomplished by simply shining an intense fs-laser pulse in the visible at normal incidence. The onset of the ultrafast transient resonance in transmission can be probed in real time as symmetry is destroyed and restored, making our theoretical proposal suitable for an experimental demonstration. Indeed, our model is realistic, as the parameters employed in the simulations are derived from experimentally validated studies; notwithstanding this, we stress that high-quality fabrication and accuracy in the measurements setup will be required to observe such effects in similar metasurfaces.

As for applications that require high resonance contrast (or, equivalently, higher pump-probe signals), we highlight that the proposed mechanism could be employed provided that the design of the metasurface and experiment is optimized. A first strategy consists in using higher levels of photoexcitation to enhance asymmetry and, hence, the in-coupling between the probe and the quasi-BIC mode. Alternatively, the geometrical degrees of freedom in the meta-atom design could be exploited to tune the BIC either at lower energies below bandgap, where residual losses are smaller, or right at the band-edge. In that specific spectral position, as experimentally demonstrated in a previous work by some of us^47^, the band-filling mechanism grants a photo-induced reduction of absorption (i.e., a negative imaginary part of Δε), possibly to the point of cancelling completely the material losses and activating gain.

Finally, we note that our proposed approach can be easily adapted to metasurfaces based on other semiconductors, in which the photoexcitation of a non-equilibrium population of carriers is efficient, including e.g. silicon, which is the most commonly employed in photonic devices.

## Methods

The numerical simulations presented in this work have been performed with the commercial application COMSOL Multiphysics 6.2^42^, employing finite-element methods for the solution of partial differential equations. We describe here both the electromagnetic eigenvalue analysis and the dynamical model (comprehending electromagnetic scattering simulations, to predict the optical response to both pump and probe pulse, as well as the I2TM integration, to describe the carrier density and lattice temperature evolution).

### Eigenvalue study

The eigenvalue problem of Maxwell’s equations in the frequency domain is solved using the Wave Optics module.

For the nanowires metasurface, the computational domain is two-dimensional; it includes the metasurface unit cell (see Fig. 1a, nanowire on substrate, surrounded by air), plus two additional external domains on top and bottom, for implementing Perfectly Matched Layers (PMLs). These constitute absorbing media, so that the problem is defined with open boundary conditions. On their outer boundary, moreover, we set scattering boundary conditions. On the *y*-edges of the unit cell, Floquet periodic boundary conditions are enforced with the wave vector ${k}_{x}\hat{{\bf{x}}}$. Instead, the out-of-plane wave vector ${k}_{z}\hat{{\bf{z}}}$ is directly incorporated in the equation formulation (in the specific option of the “physics” node, in the application). In this way, the **k**_∥_ vector is completely specified.

For the hexagonal grating, the 3D-computational domain is the hexagonal unit cell (slab with circular hole, immersed in air), plus PMLs on top and bottom, with scattering boundary conditions on the outer edges. Floquet periodicity is applied on the lateral edges, with wavevector (*k*_*x*_, *k*_*y*_).

### Dynamical model

We describe here the algorithm followed for the dynamical modelling of the nanowires metasurface and hexagonal grating. Section S2 of the SI lists tables with all the numerical values of the parameters defined below.

#### Pump

We built a full-wave scattering simulation in COMSOL, to extract the metasurface response to the pump pulse. Specifically, Maxwell’s equations in the frequency formulation are solved on a computational domain similar to the one described for the eigenvalue analysis, and corresponding to the unit cell of the metasurface. Floquet boundary conditions are imposed on the lateral edges. PMLs are no longer applied: instead, periodic ports are implemented on the top and bottom boundaries. They allow for setting the excitation with a plane wave, which, for the nanowires metasurface, is incident at 34^∘^, from the air side. The pump is polarized in-plane (see the sketch in Fig. 3a). For the hexagonal grating, instead, the pump pulse is at normal incidence and polarized in the *y*-direction. We used the simulation results as input for the I2TM as detailed below.

#### I2TM

The equations of the I2TM are reported above, Eq. (1). As mentioned, the driving term reads

$${R}_{{\rm{abs}}}({\bf{r}},t)=A\rho ({\bf{r}},{\lambda }_{{\rm{pump}}})g(t)$$

For the 2D model of the nanowires metasurface,

$$A=\frac{FP\cos ({\theta }_{{\rm{inc}}})}{{E}_{{\rm{pump}}}}$$

where *F* is the pump fluence, *P* is the width of the unit cell, θ_inc_ is the pump angle of incidence. *E*_pump_ is the energy of the single pump photon, *E*_pump_ = *hc*/*λ*_pump_, with *h* the Planck constant, *c* is the speed of light, *λ*_pump_ is the pump wavelength. *A* then represents the number of pump photons (per unit length), delivered to the sample unit cell. Instead, for the model of the hexagonal grating,

$$A=\frac{F{l}^{2}3\sqrt{3}/2\cos ({\theta }_{{\rm{inc}}})}{{E}_{{\rm{pump}}}}$$

Since ${l}^{2}3\sqrt{3}/2$ is the unit cell cross-sectional area, again, *A* represents the number of incident pump photons.

The absorption field *ρ*(**r**, *λ*_pump_) is defined as the density of dissipated power, normalized to the incident power (per unit length, in the 2D model). Thus, for the nanowires metasurface, it has dimensions of a surface density, whereas for the hexagonal grating it has dimensions of a volume density. Consistently, the dimensions of *R*_abs_ are [m^−3^ s^−1^] in both cases. The field *ρ*(**r**, *λ*_pump_) is extracted from the pump simulation, as mentioned above. The temporal dependence is instead

$$g(t)=\sqrt{\frac{4\ln 2}{\pi {\tau }_{\,\text{FWHM}\,}^{2}}}\exp \left(-4\ln 2\frac{{t}^{2}}{{\tau }_{\,\text{FWHM}\,}^{2}}\right)$$

with *τ*_FWHM_ the pulse temporal full width at half maximum.

The other parameters are the ambipolar diffusion constant *D*, the recombination time *τ*_rec_, the AlGaAs bandgap energy *E*_g_, the lattice heat capacity *c*_L_ and conductivity *κ*_L_. Their values are listed in Table 1 in section S2, along with the relevant references from which they were extracted.

The I2TM model is solved in COMSOL on the nanowire (or slab) domain within the unit cell. The “zero flux” boundary condition is enforced for both equations:

$$\begin{array}{rcl}&&{\bf{n}}\cdot \left(D{\mathbf{\nabla }}N\right)=0\\ &&{\bf{n}}\cdot \left(\frac{{\kappa }_{{\rm{L}}}}{{c}_{{\rm{L}}}}{\mathbf{\nabla }}\Theta _{\rm{L}}\right)=0\end{array}$$

with **n** the outward normal direction to the nanowire (or slab) boundaries. For the hexagonal grating, the zero-flux condition is complemented by a continuity requirement for *N* and Θ_L_ on the periodic boundaries. The temporal resolution is 25 fs.

The solutions *N*(**r**, *t*) and Θ_L_(**r**, *t*) are stored and used as inputs to calculate the local permittivity variation Δ*ε*(**r**, *t*).

#### Permittivity variation

Three effects are taken into account to compute the permittivity variations. Two of them, a Drude-like mechanism and band filling, are related to the hot carriers population, while thermo-optic variations are linked to lattice heating. Semiclassical, analytical formulations are employed to extract the three different contributions Δε_D_, Δε_BF_, Δε_TO_: they are described in detail in section 2 of the SI. The total Δε is the sum of these terms:

$$\Delta \varepsilon ({\bf{r}},t,{\lambda }_{{\rm{probe}}})=\left[\Delta {\varepsilon }_{{\rm{D}}}+\Delta {\varepsilon }_{{\rm{BF}}}+\Delta {\varepsilon }_{{\rm{TO}}}\right]({\bf{r}},t,{\lambda }_{{\rm{probe}}})$$

Notice that it is a function of space and time (being dependent on both *N* and Θ_L_) and of the probe wavelength *λ*_probe_. It is used as input for simulating the sample response to a spectrally dispersed probe, arriving at selected time delay with respect to the pump peak.

#### Probe

Finally, we performed full-wave scattering simulations in COMSOL, to model the interaction between the probe pulse (wavelength *λ*_probe_, arriving at a time delay *t*) and the metasurface. This last step is almost identical to the first, i.e., the pump-related simulation. Maxwell’s equation are solved on the unit cell, with Floquet boundary conditions and periodic ports. This time, the plane-wave excitation is set at normal incidence, with *z*-polarization for the nanowires metasurface, and *x*-polarization for the hexagonal grating. For each *λ*_probe_ and delay time *t*, the permittivity is assigned locally within the nanowire, as

$$\varepsilon ({\bf{r}},t,{\lambda }_{{\rm{probe}}})={\varepsilon }_{{\rm{AlGaAs}}}+\Delta \varepsilon ({\bf{r}},t,{\lambda }_{{\rm{probe}}})$$

The spectral resolution is 0.01 nm across the whole simulated range for the calculations shown in Fig. 4. For the hexagonal grating (Fig. 6c), the resolution is finer (0.01 nm) between 768.6 nm and 768.9 nm, and coarser (0.1 nm) elsewhere. Thus, we obtain the transmission *T*(*λ*_probe_, *t*), from which it is possible to compute Δ*T*/*T* directly.

## Supplementary information

Supplemental Material

## Data Availability

All the data supporting this study are available upon request to the corresponding authors.
