# Supplementary material for: Ultrafast switching of a metasurface quasi-bound state in the continuum via transient optical symmetry breaking
Source: Light Sci Appl. 2025 Jul 8;14:240. doi: 10.1038/s41377-025-01885-z (PMC12234965; doi:10.1038/s41377-025-01885-z)
Supplement: Supplementary file 1 — Supplemental Material [file 41377_2025_1885_MOESM1_ESM.pdf]

Supporting Information for "Ultrafast switching of a metasurface quasi-bound state in the continuum via transient optical symmetry breaking"

Giulia Crotti,[*,1] Andrea Schirato,[1,2] Olesiya Pashina,[3,4] Olga Sergaeva,[4] Mihail Petrov,[3] Costantino De Angelis,[4,5] and Giuseppe Della Valle[*,1,6]

1 *Dipartimento di Fisica - Politecnico di Milano, Piazza Leonardo da Vinci, 32, I-20133 Milano, Italy*

2*Department of Physics and Astronomy, Rice University, Houston, Texas 77005, United States*

3 *School of Physics and Engineering, ITMO University, 191002 Saint Petersburg, Russia*

4 *Dipartimento di Ingegneria dell'informazione, University of Brescia, Via Branze 38, 25123 Brescia, Italy*

5 *Istituto Nazionale di Ottica, Consiglio Nazionale delle Ricerche, Via Branze, 45, Brescia, 25123, Italy*

6 *Istituto Nazionale di Fisica Nucleare – Sezione di Milano, Via Celoria 16, 20133 Milano, Italy*

E-mail: giulia1.crotti@polimi.it; giuseppe.dellavalle@polimi.it

S1 Topological description

S1.1 Nanowires metasurface

The designed metasurface (refer to Fig. 1a of the main text) is an array of nanowires of finite thickness h, with 1D-periodicity along the x-axis and continuity along the z-direction (again, consistent with the notation in Fig. 1a). It is embedded in the environment constituted by air, and supported by a substrate; both media are extended indefinitely along the y-axis direction. A resonant electromagnetic mode of this system can be written, thanks to Bloch theorem, as[1,2]

$$\mathbf{E}_{\mathbf{k}_\parallel}(x, y, z) = \mathbf{u}_{\mathbf{k}_\parallel}(x, y)e^{\mathrm{i}k_x x}e^{\mathrm{i}k_z z}$$

with $\mathbf{k}_\parallel = (k_x, k_z)$ the in-plane wavevector and $\mathbf{u}_{\mathbf{k}_\parallel}$ a periodic function of x with the same periodicity as the metasurface. This constitutes the eigensolution of Maxwell's equations for the system with open boundary conditions, with eigenvalue $\Omega(\mathbf{k}_\parallel) = \omega + \mathrm{i}\gamma$. Outside the metasurface, in the substrate and environment, the field is constituted by either propagative or evanescent waves, decaying exponentially with y. Below the diffraction limit and above the light line, the only propagative component is characterized by the in-plane wavevector $\mathbf{k}_\parallel$ and polarization $\mathbf{c}^{\mathrm{u,d}}(\mathbf{k}_\parallel) = c_x^{\mathrm{u,d}}\hat{\mathbf{x}} + c_y^{\mathrm{u,d}}\hat{\mathbf{y}} + c_z^{\mathrm{u,d}}\hat{\mathbf{z}}$, with u, d for the upward and downward directions, and[2,3]

$$\begin{cases} c_x^{\mathrm{u,d}} &= \left\langle \mathbf{u}_{\mathbf{k}_\parallel} \right\rangle_{\mathrm{u,d}} \cdot \hat{\mathbf{x}} \\ c_y^{\mathrm{u,d}} &= \left\langle \mathbf{u}_{\mathbf{k}_\parallel} \right\rangle_{\mathrm{u,d}} \cdot \hat{\mathbf{y}} \\ c_z^{\mathrm{u,d}} &= \left\langle \mathbf{u}_{\mathbf{k}_\parallel} \right\rangle_{\mathrm{u,d}} \cdot \hat{\mathbf{z}} \end{cases}$$

The brackets $\langle \cdot \rangle_{\mathrm{u,d}}$ indicate the spatial average over a given xz plane above or below the unit cell. Indeed, $\mathbf{c}^{\mathrm{u,d}}(\mathbf{k}_\parallel)$ corresponds to the zero-order Fourier coefficient of $\mathbf{u}_{\mathbf{k}_\parallel}$.

To discuss the topology of the system, we define the two-dimensional projection $\mathbf{c}'^{\mathrm{u,d}}(\mathbf{k}_\parallel)$ of $\mathbf{c}^{\mathrm{u,d}}(\mathbf{k}_\parallel)$ onto the xz plane, following ref. 2: $\mathbf{c}'^{\mathrm{u,d}}(\mathbf{k}_\parallel) = c_x^{\mathrm{u,d}}\hat{\mathbf{x}} + c_z^{\mathrm{u,d}}\hat{\mathbf{z}}$. This is the so-called far-field polarization. The maps of Figs. 1e and 2b in the main document are obtained

by representing, for each $\mathbf{k}_\parallel$, the main axis of the polarization ellipse described by $\mathbf{c}'^{\mathrm{d}}(\mathbf{k}_\parallel)$. Notice that the singularities (i.e., points in the reciprocal space where this vector field is not well defined) correspond to *loci* where either the modulus of $\mathbf{c}'^{\mathrm{d}}(\mathbf{k}_\parallel)$ vanishes (downward radiation is forbidden) or the polarization is circular (C-points).

The charge of such topological defects is defined as[2,3]

$$q = \frac{1}{2\pi}\oint_C \mathrm{d}\mathbf{k}_\parallel \cdot \nabla_{\mathbf{k}_\parallel}\phi(\mathbf{k}_\parallel)$$

with C a closed path encircling the singularity, travelled counterclockwise,

$$\phi(\mathbf{k}_\parallel) = \frac{1}{2}\arg\left[S_1(\mathbf{k}_\parallel) + \mathrm{i}S_2(\mathbf{k}_\parallel)\right],$$

and S_1, S_2 are the Stokes parameters of $\mathbf{c}'^{\mathrm{d}}(\mathbf{k}_\parallel)$ (or $\mathbf{c}'^{\mathrm{u}}(\mathbf{k}_\parallel)$, depending on the space under scrutiny). The definition is well-posed since $\mathbf{u}_{\mathbf{k}_\parallel}$ is chosen as a smooth function of $\mathbf{k}_\parallel$. In this way, ϕ represents the angle between the main axis of the polarization ellipse and the horizontal axis, and q is the number of times the polarization vector winds along the loop. BICs have $S_1 = S_2 = S_3 = 0$ and integer q,[2–6] whereas C-points have $S_1 = S_2 = 0$, $S_3 = \pm 1$ and semi-integer q.[3,7–9]

Notes on symmetry. Notice that the simulated 1D-periodic metasurface does *not* have a mirror symmetry with respect to the horizontal xz plane (i.e., it is not σ_y symmetric), either in the unperturbed or perturbed condition. As such, the outgoing waves on one side do not determine the outgoing waves on the other side. Consequently, a topological defect with integer charge, e.g., in the downward far-field polarization, is in general only a necessary, but not sufficient condition for the presence of a BIC: it may represent a point of reciprocal space in which only upward radiation is allowed. This phenomenon has been exploited for creating the so-called unidirectional guided resonances.[9] However, in our specific case, the topological defect with charge $q = -1$ in unperturbed conditions is located at Γ, namely at

a high-symmetry point for the structure. There, the C_2^y symmetry implies that if $\mathbf{c}'^{\mathrm{d}}(\mathbf{k}_\parallel)$ vanishes, then also $\mathbf{c}'^{\mathrm{u}}(\mathbf{k}_\parallel)$ should vanish, and viceversa. In other words, in these high-symmetry points, BICs are stable even when no additional mirror symmetry is present.[2]

As mentioned in the main text, the in-plane symmetries of the metasurface are the elements of the $C_{2\mathrm{v}}$ group. Namely, beside C_2^y, the structure is also symmetric with respect to the mirror reflections σ_v about the vertical xy and yz planes. The studied at-Γ BIC is symmetry-protected since it belongs to a different symmetry class with respect to propagative waves at normal incidence: indeed, it is even under C_2^y, while plane waves are odd. More specifically, one could say that this BIC belongs to the A_2 representation of $C_{2\mathrm{v}}$, being even under C_2^y and odd under both $\sigma_\mathrm{v}(xy)$ and $\sigma_\mathrm{v}(yz)$. Instead, z polarized plane waves at normal incidence are odd under C_2^y and $\sigma_\mathrm{v}(xy)$, but even under $\sigma_\mathrm{v}(yz)$. Then, z polarized plane waves belong to the B_2 representation of $C_{2\mathrm{v}}$. From similar considerations, x polarized plane waves belong to the B_1 representation.

Since C_2^y symmetry corresponds to the composition of $\sigma_\mathrm{v}(xy)$ and $\sigma_\mathrm{v}(yz)$, a perturbation that breaks it necessarily destroys at least one of the mirror symmetries. In our case, the pump-induced perturbation breaks C_2^y and $\sigma_\mathrm{v}(yz)$, while maintaining $\sigma_\mathrm{v}(xy)$. Then, consistently, the at-Γ BIC turns into a quasi-BIC leaky mode coupled to z polarized plane waves.

S1.2 Hexagonal grating

A similar discussion applies for the 2D-periodic grating with hexagonal unit cell presented in the main text (refer to Figs. 5 and 6), in which a resonance can be written as

$$\mathbf{E}_{\mathbf{k}_\parallel}(x, y, z) = \mathbf{u}_{\mathbf{k}_\parallel}(x, y, z) e^{\mathrm{i}k_x x} e^{\mathrm{i}k_y y}$$

This time, since z is the coordinate in the normal direction to the slab, the in-plane wavevector is $\mathbf{k}_\parallel = (k_x, k_y)$ and $\mathbf{u}_{\mathbf{k}_\parallel}$ is a periodic function in $x\hat{\mathbf{x}} + y\hat{\mathbf{y}}$. Here, $\mathbf{c}'^{\mathrm{u,d}}(\mathbf{k}_\parallel)$ is the projection

of the polarization onto the xy plane, $\mathbf{c}'^{\mathrm{u,d}}(\mathbf{k}_\parallel) = c_x^{\mathrm{u,d}}\hat{\mathbf{x}} + c_y^{\mathrm{u,d}}\hat{\mathbf{y}}$, with $c_x^{\mathrm{u,d}}$ and $c_y^{\mathrm{u,d}}$ defined as above, as the spatial average of $\mathbf{u}_{\mathbf{k}_\parallel}$ over some xy plane above or below the unit cell, projected along the x and y directions.

Notes on symmetry. In this case, in unperturbed conditions, the structure is mirror symmetric with respect to the xy plane (i.e., it is σ_z symmetric). Hence, an integer-charged singularity in the downward or upward far-field polarization is a necessary and sufficient condition for the presence of a BIC. However, σ_z is destroyed by photo-excitation, as well as C_3 symmetry. Thus, the $q = -1$ topological singularities appearing either in the upward or downward far-field polarization upon the splitting of the at-Γ BIC, after pump arrival, are not BICs. Rather, they are points in reciprocal space where either upward or downward radiation is (separately) forbidden. This can be seen clearly by inspecting the polarization fields relative to both $\mathbf{c}'^{\mathrm{u}}(\mathbf{k}_\parallel)$ and $\mathbf{c}'^{\mathrm{d}}(\mathbf{k}_\parallel)$ at $t = 300\,\mathrm{fs}$, as shown in Fig. S1a (red and blue curves, respectively). The $q = -1$ singularities on the k_x-axis are marked with dots of the same colours. Indeed, they are not superimposed, so that it does not exist a point in reciprocal space where upward and downward radiation is simultaneously forbidden.

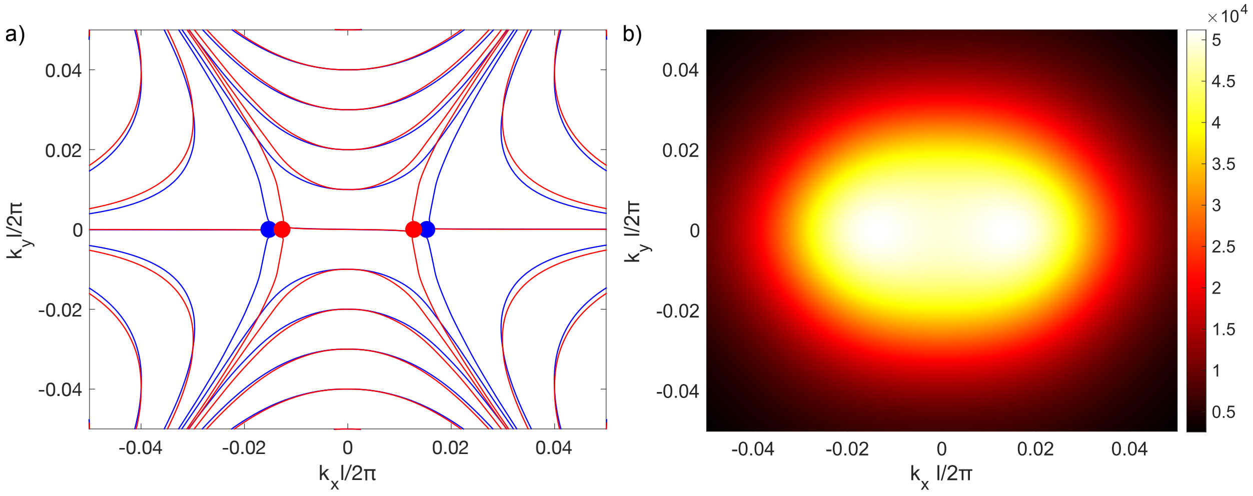

Figure S1: (a): far-field polarization in the upward (red) and downward (blue) direction at $t = 300\,\mathrm{fs}$. (b): quality factor of the photonic band at $t = 300\,\mathrm{fs}$. Notice that the color scale is linear.

This is reflected also by the quality factor of the resonances across the photonic band, as

shown in fig. S1b. There are no divergences, even though the quality factor is high in the singularities' region on the k_x-axis.

As mentioned in the main text, the $C_{6\mathrm{v}}$ point group describes the in-plane symmetries of this metasurface. Indeed, beside C_6, C_3 and C_2, the other symmetries are (i) three mirror reflections σ_d (about the planes spanned by the hexagon diagonals and the z axis), and (ii) three mirror reflections σ_v (about the planes spanned by the in-plane lines bisecting two opposite sides, and the z axis). In this case, the BIC mode can be said to belong to the B_1 representation of $C_{6\mathrm{v}}$, being odd under C_2 and C_6, even under C_3, even under σ_v and odd under σ_d.

The pump-induced perturbation breaks C_3 (and also, necessarily, C_6, two of the three mirror symmetries σ_d and two of the three mirror symmetries σ_v). On the other hand, C_2 is left intact, as well as the mirror symmetries about the xz and yz planes, for which the BIC is even and odd, respectively. This means that the BIC switches to a quasi-BIC in the B_1 representation of the $C_{2\mathrm{v}}$ group. This quasi-BIC mode, consistently, can be excited by a probe constituted by an x polarized plane wave propagating at normal incidence.

S2 Modelling of the ultrafast photoexcitation

We report here additional information on our model of the structure nonequilibrium response. Specifically, we first discuss some of the parameters employed for the I2TM (see table S1). In a second paragraph, we describe in detail how we computed the photo-induced permittivity variation, providing the values of the necessary material parameters in a dedicated table (S2).

I2TM. The lattice parameters are estimated considering an Al percentage of 18%, whereas the bandgap energy corresponds to the one computed for Al 18.5%, as in a recent work by some of the present authors.[14]

The ambipolar diffusion parameter D is treated as an effective parameter and estimated

Table S1: Parameters for the I2TM.

Parameter	Description	Value	Ref.
F	Pump fluence	$42.22\,\mu J\,cm^{-2}$ (nanowires)<br>$70\,\mu J\,cm^{-2}$ (hexagonal grating)	–
P	Unit cell width (periodicity)	400 nm	–
θ_{inc}	Pump angle of incidence	$34°$ (nanowires)<br>$0°$ (hexagonal grating)	–
λ_{pump}	Pump wavelength	400 nm	–
τ_{FWHM}	Pump full width at half maximum	100 fs	–
D	Ambipolar diffusion constant	$18\,cm^2\,s^{-1}$	10–14
τ_{rec}	Trap-assisted recombination time	8 ps	12,14
E_g	AlGaAs bandgap energy	1.65 eV	15
c_L	Lattice heat capacity	$1.77 \times 10^6\,J\,m^{-3}\,K^{-1}$	15
κ_L	Lattice conductivity	$24.88\,W\,m^{-1}\,K^{-1}$	16

by considering the measured value for bulk GaAs at room temperature,[10,11] in agreement with previous reports on AlGaAs,[12–14] where reduced diffusion models were able to reproduce experimental results quantitatively.

Permittivity variation. Three effects are taken into account to compute the permittivity variations. Two of them, a Drude-like mechanism and band filling, are related to the hot carriers population, while thermo-optic variations are linked to lattice heating.

The Drude contribution accounts for the opening of channels for intraband transitions. The pump-induced populations of electron and holes behave as Drude plasmas. The permittivity variation $\Delta\varepsilon_D = \mathrm{Re}(\Delta\varepsilon_D) + i\mathrm{Im}(\Delta\varepsilon_D)$ is given by the expressions

$$\begin{aligned} \mathrm{Re}(\Delta\varepsilon_D) &= -\frac{e^2 N}{m^* \varepsilon_0 \left[(2\pi c/\lambda_{probe})^2 + \Gamma_D^2\right]} \\ \mathrm{Im}(\Delta\varepsilon_D) &= -\mathrm{Re}(\Delta\varepsilon_D)\frac{\lambda_{probe}\Gamma_D}{2\pi c} \end{aligned} \tag{1}$$

Notice that we have left the temporal and spatial dependence implicit, for the sake of readability; in our model, the permittivity variation is local, and defined at a selected time delay and probe wavelength. In these formulae, e is the electron charge, ε_0 is the vacuum permit-

tivity, c is the speed of light. Moreover, Γ_D is the Drude damping coefficient, while m^* is the plasma reduced mass, defined as

$$m^* = \left(\frac{1}{m_e} + \frac{1}{m_h^*}\right)^{-1}$$

in turn, m_e and m_h^* are the electron and hole effective masses, with m_h^* defined considering the contribution of both light and heavy holes:

$$m_h^* = \frac{m_{lh}^{3/2} + m_{hh}^{3/2}}{m_{lh}^{1/2} + m_{hh}^{1/2}}$$

The values of these parameters are reported in table S2. Here, Γ_D is chosen in agreement with refs. 12,14, in which similar models were validated on experimental results; the effective masses are calculated according to literature[15] for an Al percentage of 18%.

The band filling effect[17] considers the modulation of the interband transitions. The computation follows the steps outlined in refs.[12–14,18] The pump-induced electron-hole pairs occupy, respectively, the lowest and highest part of the conduction and valence band, leading to a saturation of absorption α for wavelengths shorter than the bandgap wavelength λ_g. Using the parabolic bands approximation, in the vicinity of the bandgap, the modulation of α reads

$$\Delta\alpha = \begin{cases} \frac{\lambda_{probe}}{\sqrt{c}}\sqrt{\frac{1}{\lambda_{probe}} - \frac{1}{\lambda_g}}\left[C_{lh}G_{lh}(\lambda_{probe}, N) + C_{hh}G_{hh}(\lambda_{probe}, N)\right] & \lambda_{probe} \leq \lambda_g \\ 0 & \lambda_{probe} > \lambda_g \end{cases}$$

The functions G_{lh}, G_{hh}, incorporating the N-dependent quasi-Fermi level for the conduction and valence bands, are calculated as detailed in ref.[14] with the parameters listed in Table S2. The values of C_{lh}, C_{hh}, which are dependent on the material properties, are also included in the same table. The modulation $\Delta\alpha$ is related to the modification of the material refractive

index $n_{\mathrm{AlGaAs}} + \mathrm{i}k_{\mathrm{AlGaAs}} = \sqrt{\varepsilon_{\mathrm{AlGaAs}}}$ by

$$\Delta k_{\mathrm{BF}} = \lambda_{\mathrm{probe}} \frac{\Delta \alpha}{2}$$

Via Kramers-Kronig relations, the contribution to the real part modulation Δn_{BF} can be derived. In contrast with Δk_{BF}, Δn_{BF} is non-vanishing also for $\lambda_{\mathrm{probe}} > \lambda_{\mathrm{g}}$. To the first order in Δn_{BF}, Δk_{BF}, the permittivity variation due to band filling is derived as

$$\begin{aligned} \mathrm{Re}(\Delta\varepsilon_{\mathrm{BF}}) &= 2[n_{\mathrm{AlGaAs}}\Delta n_{\mathrm{BF}} - k_{\mathrm{AlGaAs}}\Delta k_{\mathrm{BF}}] \\ \mathrm{Im}(\Delta\varepsilon_{\mathrm{BF}}) &= 2[n_{\mathrm{AlGaAs}}\Delta k_{\mathrm{BF}} + k_{\mathrm{AlGaAs}}\Delta n_{\mathrm{BF}}] \end{aligned} \tag{2}$$

Again, note that explicit mention of spatio-temporal dependency has been omitted in the expressions for deriving $\Delta\varepsilon_{\mathrm{BF}}$. Moreover, it is worth highlighting that, in the spectral region of interest where $\lambda_{\mathrm{probe}} > \lambda_{\mathrm{g}}$, $\Delta\varepsilon_{\mathrm{BF}}$ is purely real.

Lastly, the thermo-optic contribution $\Delta\varepsilon_{\mathrm{TO}}$, induced by the increase in lattice temperature $\Delta\Theta_{\mathrm{L}}$, is computed starting from the thermo-optic coefficient η. This is defined phenomenologically as $\eta = \partial(n_{\mathrm{AlGaAs}})/\partial\Theta_{\mathrm{L}}$. Thus, for a small increase in Θ_{L}, $\Delta n_{\mathrm{TO}} = \eta\Delta\Theta_{\mathrm{L}}$. To the first order in $\Delta\Theta_{\mathrm{L}}$, then,

$$\begin{aligned} \mathrm{Re}(\Delta\varepsilon_{\mathrm{TO}}) &= 2n_{\mathrm{AlGaAs}}\eta\Delta\Theta_{\mathrm{L}} \\ \mathrm{Im}(\Delta\varepsilon_{\mathrm{TO}}) &= 2k_{\mathrm{AlGaAs}}\eta\Delta\Theta_{\mathrm{L}} \end{aligned} \tag{3}$$

As shown in ref.,[19] η is in general dependent on λ_{probe}. For simplicity, we chose to employ a fixed constant (which can be found in Table S2), as in ref.,[14] and we neglected $\Delta\Theta_{\mathrm{L}}$-related variations of k_{AlGaAs}. The total permittivity variation is the sum of all three contributions:

$$\Delta\varepsilon(\mathbf{r}, t, \lambda_{\mathrm{probe}}) = [\Delta\varepsilon_{\mathrm{D}} + \Delta\varepsilon_{\mathrm{BF}} + \Delta\varepsilon_{\mathrm{TO}}]\,(\mathbf{r}, t, \lambda_{\mathrm{probe}})$$

We stress that, as our model is temporally segregated and we maintain the hypothesis of

Table S2: Parameters for $\Delta\varepsilon_{\mathrm{D}}$, $\Delta\varepsilon_{\mathrm{BF}}$ and $\Delta\varepsilon_{\mathrm{TO}}$.

Parameter	Description	Value	Ref.
Γ_{D}	Damping constant	$4.11 \times 10^{13}\,\mathrm{s}^{-1}$	14
m_{e}	Electron effective mass	$7.10 \times 10^{-32}\,\mathrm{kg}$	15
m_{lh}	Light hole effective mass	$8.58 \times 10^{-32}\,\mathrm{kg}$	15
m_{hh}	Heavy hole effective mass	$5.06 \times 10^{-31}\,\mathrm{kg}$	15
C_{lh}	Light holes constant	$3.85 \times 10^{13}\,\mathrm{m}^{-1}\,\mathrm{s}^{-0.5}$	13,14
C_{hh}	Heavy holes constant	$7.81 \times 10^{13}\,\mathrm{m}^{-1}\,\mathrm{s}^{-0.5}$	13,14
η	Thermo-optic coefficient	$1.00 \times 10^{-3}\,\mathrm{K}^{-1}$	19

linear pump absorption, we neglect all photo-induced modulations at the pump wavelength (i.e., pump self-effects are overlooked). Hence, the $\Delta\varepsilon$ above is computed for each probe wavelength and exclusively taken into account for the probe interaction. This approach has been experimentally validated for low to moderate levels of photo-excitation, such as the ones employed in this work[14,18].

As a visual reference, Fig. S2 shows the real and imaginary part of $\Delta\varepsilon$ in the spectral range 760 nm – 800 nm, evaluated for three different values of the electron-hole pair density. The data are computed setting $\Delta\Theta_{\mathrm{L}} = 0.5\,\mathrm{K}$.

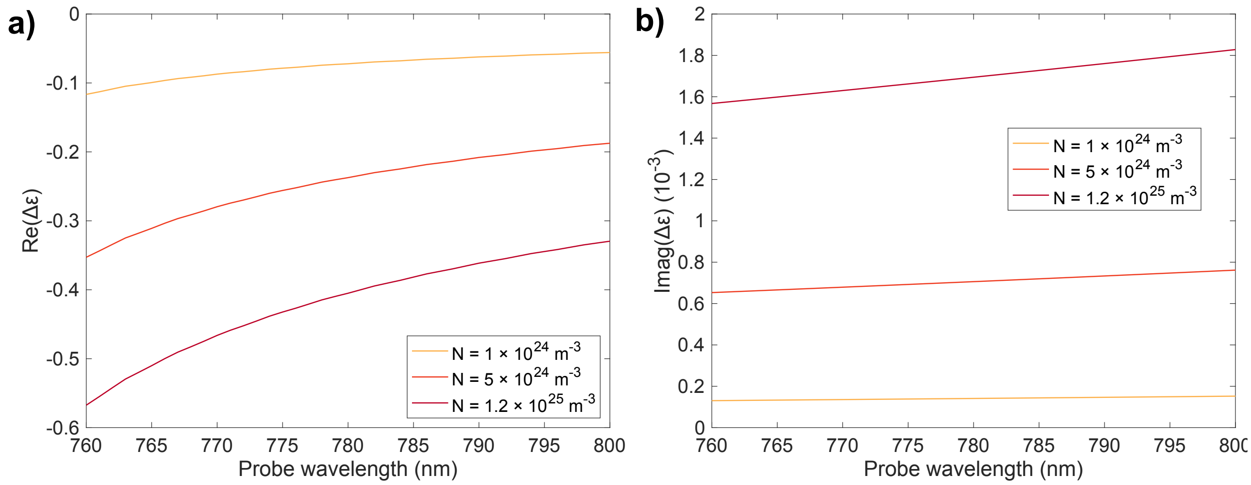

Figure S2: Examples of typical $\Delta\varepsilon$ spectra. a) Real part of the permittivity variation, as a function of probe wavelength, computed for different values of the electron-hole pairs N (increasing from lighter to darker shades, see the legend). b) Same as a) for the imaginary part of permittivity variation.

S3 Pump absorption and induced asymmetry

In this section, we add further details on the pump absorption process, explaining the choice of the excitation conditions (pump energy and angle of incidence) that we considered in the numerical experiment on the nanowire metasurface, and their impact on the optically-induced asymmetries.

Our model of AlGaAs photoexcitation is based on the assumption of linear absorption. This applies if pumping above bandgap, in a spectral region where the imaginary part of the refractive index is high. Thus, the selected pump wavelength is 400 nm. At 400 nm, the meta-atoms are smaller than the wavelength, yet optically dense, and the material skin-depth is one order of magnitude smaller than their typical size.[12–14] Hence, it is possible to attain an inhomogeneous photoexcitation at the individual meta-atom scale, even under illumination which is spatially uniform over the typical spot-size of a beam.[20] This is consistent with the results of our simulations (see Fig. 3a of the main text), where the pump excitation is modelled as a plane wave impinging on an infinitely extended metasurface.

These assumptions (plane wave, infinite periodic medium) imply that our model is accurate as long as the pump spot size, in a real experiment, is sufficiently large to illuminate hundreds of meta-atoms in an approximately uniform fashion. In our case, this condition is satisfied if the (Gaussian) beam intensity has a spatial full width at half maximum of $\sim 100\,\mu\mathrm{m}$.

For the nanowires metasurface, the pump angle of incidence was optimized to meet two criteria: experimental feasibility and maximization of the left-right asymmetry in terms of absorption. To this aim, we performed a preliminary parametric exploration, simulating the pump excitation with different angles of incidence in the range 20° - 50° (where 0° is normal

incidence). We defined the following figure of merit for the absorption asymmetry:

$$Q_{\text{asymm}} = \frac{\int_{\text{L}} d\mathbf{r}\; q(\mathbf{r}) - \int_{\text{R}} d\mathbf{r}\; q(\mathbf{r})}{\int_{\text{wire}} d\mathbf{r}\; q(\mathbf{r})} \tag{4}$$

where $q(\mathbf{r})$ are the electromagnetic losses (i.e., the density of dissipated power), $\int_{\text{wire}}$ denotes the spatial integral defined on the nanowire in the unit cell, while $\int_{\text{L}}$ and $\int_{\text{R}}$ are the integrals relative to its left and right half, respectively. Q_{asymm} is plotted in figure S3a as a function of the angle of incidence. While its maximum is at 34°, which was the selected angle for the metasurface excitation, all the angles between $\sim 30°$ and $\sim 45°$ are expected to yield similarly good results in terms of optical symmetry breaking.

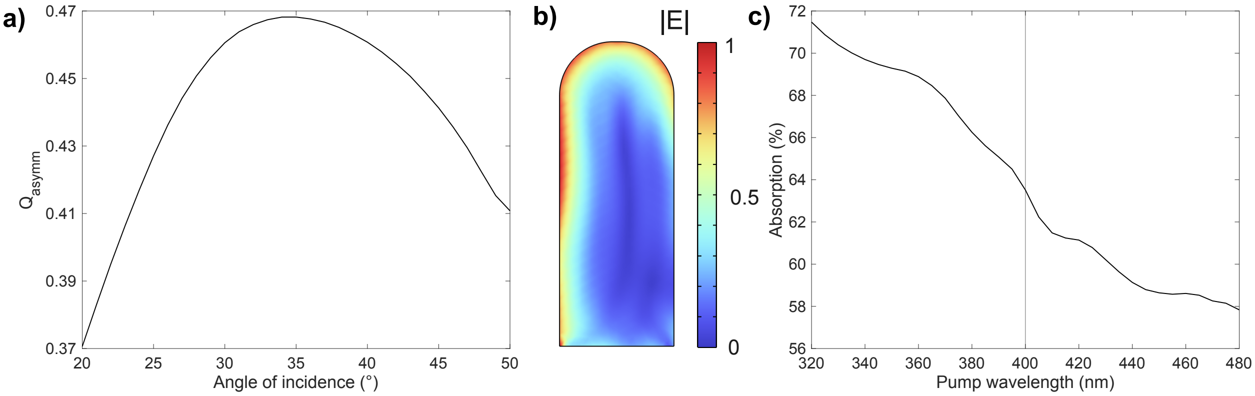

Figure S3: Details on the pump excitation. a) Figure of merit for the asymmetric pump absorption, as a function of the angle of incidence. b) Norm of the electric field induced inside the nanowire by the pump ($\lambda_{\text{pump}} = 400\,\text{nm}$, $\theta_{\text{inc}} = 34°$), normalized to its maximum value. c) Absorption spectrum for $\theta_{\text{inc}} = 34°$. The vertical line at 400 nm corresponds to the selected value of pump wavelength.

Fig. S3b shows the norm of the electric field (normalized to its maximum) induced by the pump ($\lambda_{\text{pump}} = 400\,\text{nm}$, $\theta_{\text{inc}} = 34°$) inside the nanowire. As expected, its spatial profile corresponds to the distribution of electromagnetic losses, thus to the $\rho(\mathbf{r}, \lambda_{\text{pump}})$ field depicted in Fig. 3a of the main text.

For completeness, Fig. S3c shows the absorption spectrum for $\theta_{\text{inc}} = 34°$. The selected wavelength (vertical line in the graph) corresponds to a non-resonant pumping condition.

References

(1) Joannopoulos, J. D.; Johnson, S. G.; Winn, J. N.; Meade, R. D. *Photonic Crystals: Molding the Flow of Light - Second Edition*; Princeton University Press, 2011.

(2) Zhen, B.; Hsu, C. W.; Lu, L.; Stone, A. D.; Soljačić, M. Topological Nature of Optical Bound States in the Continuum. *Physical Review Letters* **2014**, *113*.

(3) Yoda, T.; Notomi, M. Generation and Annihilation of Topologically Protected Bound States in the Continuum and Circularly Polarized States by Symmetry Breaking. *Physical Review Letters* **2020**, *125*.

(4) Hsu, C. W.; Zhen, B.; Stone, A. D.; Joannopoulos, J. D.; Soljačić, M. Bound states in the continuum. *Nature Reviews Materials* **2016**, *1*.

(5) Doeleman, H. M.; Monticone, F.; den Hollander, W.; Alù, A.; Koenderink, A. F. Experimental observation of a polarization vortex at an optical bound state in the continuum. *Nature Photonics* **2018**, *12*, 397–401.

(6) Jin, J.; Yin, X.; Ni, L.; Soljačić, M.; Zhen, B.; Peng, C. Topologically enabled ultrahigh-Q guided resonances robust to out-of-plane scattering. *Nature* **2019**, *574*, 501–504.

(7) Zhou, H.; Peng, C.; Yoon, Y.; Hsu, C. W.; Nelson, K. A.; Fu, L.; Joannopoulos, J. D.; Soljačić, M.; Zhen, B. Observation of bulk Fermi arc and polarization half charge from paired exceptional points. *Science* **2018**, *359*, 1009–1012.

(8) Liu, W.; Wang, B.; Zhang, Y.; Wang, J.; Zhao, M.; Guan, F.; Liu, X.; Shi, L.; Zi, J. Circularly Polarized States Spawning from Bound States in the Continuum. *Physical Review Letters* **2019**, *123*.

(9) Yin, X.; Jin, J.; Soljačić, M.; Peng, C.; Zhen, B. Observation of topologically enabled unidirectional guided resonances. *Nature* **2020**, *580*, 467–471.

(10) Ruzicka, B. A.; Werake, L. K.; Samassekou, H.; Zhao, H. Ambipolar diffusion of photoexcited carriers in bulk GaAs. *Applied Physics Letters* **2010**, *97*.

(11) Chen, K.; Sheehan, N.; He, F.; Meng, X.; Mason, S. C.; Bank, S. R.; Wang, Y. Measurement of Ambipolar Diffusion Coefficient of Photoexcited Carriers with Ultrafast Reflective Grating-Imaging Technique. *ACS Photonics* **2017**, *4*, 1440–1446.

(12) Mazzanti, A.; Pogna, E. A. A.; Ghirardini, L.; Celebrano, M.; Schirato, A.; Marino, G.; Lemaítre, A.; Finazzi, M.; De Angelis, C.; Leo, G.; Cerullo, G.; Della Valle, G. All-Optical Modulation with Dielectric Nanoantennas: Multiresonant Control and Ultrafast Spatial Inhomogeneities. *Small Science* **2021**, *1*.

(13) Pogna, E. A. A.; Celebrano, M.; Mazzanti, A.; Ghirardini, L.; Carletti, L.; Marino, G.; Schirato, A.; Viola, D.; Laporta, P.; De Angelis, C.; Leo, G.; Cerullo, G.; Finazzi, M.; Della Valle, G. Ultrafast, All Optically Reconfigurable, Nonlinear Nanoantenna. *ACS Nano* **2021**, *15*, 11150–11157.

(14) Crotti, G.; Akturk, M.; Schirato, A.; Vinel, V.; Trifonov, A. A.; Buchvarov, I. C.; Neshev, D. N.; Proietti Zaccaria, R.; Laporta, P.; Lemaître, A.; Leo, G.; Cerullo, G.; Maiuri, M.; Della Valle, G. Giant ultrafast dichroism and birefringence with active nonlocal metasurfaces. *Light: Science & Applications* **2024**, *13*.

(15) Levinshtein, M.; Rumyantsev, S.; Shur, M. *Handbook Series on Semiconductor Parameters: In 2 Volumes*; WORLD SCIENTIFIC, 1996.

(16) Afromowitz, M. A. Thermal conductivity of Ga1-xAlxAs alloys. *Journal of Applied Physics* **1973**, *44*, 1292–1294.

(17) Bennett, B.; Soref, R.; Del Alamo, J. Carrier-induced change in refractive index of InP, GaAs and InGaAsP. *IEEE Journal of Quantum Electronics* **1990**, *26*, 113–122.

(18) Shcherbakov, M. R.; Liu, S.; Zubyuk, V. V.; Vaskin, A.; Vabishchevich, P. P.; Keeler, G.; Pertsch, T.; Dolgova, T. V.; Staude, I.; Brener, I.; Fedyanin, A. A. Ultrafast all-optical tuning of direct-gap semiconductor metasurfaces. *Nature Communications* **2017**, *8*.

(19) Celebrano, M. et al. Optical tuning of dielectric nanoantennas for thermo-optically reconfigurable nonlinear metasurfaces. *Optics Letters* **2021**, *46*, 2453.

(20) Schirato, A.; Crotti, G.; Proietti Zaccaria, R.; Alabastri, A.; Della Valle, G. Hot carrier spatio-temporal inhomogeneities in ultrafast nanophotonics. *New Journal of Physics* **2022**, *24*, 045001.
